# Increased Dickkopf-1 in Recent-onset Rheumatoid Arthritis is a New Biomarker of Structural Severity. Data from the ESPOIR Cohort

**DOI:** 10.1038/srep18421

**Published:** 2016-01-20

**Authors:** Raphaèle Seror, Saida Boudaoud, Stephan Pavy, Gaetane Nocturne, Thierry Schaeverbeke, Alain Saraux, Philippe Chanson, Jacques-Eric Gottenberg, Valérie Devauchelle-Pensec, Gabriel J. Tobón, Xavier Mariette, Corinne Miceli-Richard

**Affiliations:** 1 Service de Rhumatologie–Hôpitaux Universitaires Paris Sud, Le Kremlin Bicêtre, France; 2 Unité INSERM U1012 - Université Paris Sud, Le Kremlin Bicêtre, France; 3 Groupe Hospitalier Pellegrin, Bordeaux, France; 4 CHU de la Cavale Blanche, Brest Cedex, France; 5 Assistance-Publique- Hôpitaux de Paris, Service d’Endocrinologie et des Maladies de la Reproduction, - Hôpitaux Universitaires Paris Sud, F-94275, Le Kremlin Bicêtre, France; 6 Univ Paris-Sud, UMR S693, F-94276, Le Kremlin Bicêtre, France; 7 Service de Rhumatologie, Centre National de Référence pour les Maladies Auto-Immunes Systémiques Rares, Hôpital de Hautepierre, Hôpitaux Universitaires de Strasbourg, Strasbourg, France

## Abstract

Rheumatoid arthritis (RA) is the most common chronic inflammatory rheumatic condition over the world. RA is potentially disabling because chronic inflammation of the joints leads to joint destruction. To date, the best predictor of radiographic progression for patients with early RA is the presence of radiographic erosions at baseline, but a limited number of predictive biomarkers of structural progression are currently used in daily practice. Here, we investigated Dickkopf-1 (DKK-1) and sclerostin (SOST) serum levels in patients with recent inflammatory arthritis from the ESPOIR cohort. This cohort is a prospective, multicenter French cohort of 813 patients with early arthritis. We observed that mean baseline DKK-1 level was higher among RA patients with than without radiological progression within the first 2 years of evolution. DKK-1 level was still associated with radiographic progression in a model including other main predictors of severity (erosions at baseline, and anti-CCP antibody positivity). This study demonstrates that increased DKK-1 level at baseline predicted structural progression after 2-year follow-up and suggests that DKK-1 might be a new structural biomarker for early RA.

Rheumatoid arthritis (RA) is the most common chronic inflammatory rheumatic condition; the prevalence ranges from 0.5% to 1% in the population. The hallmark of RA is synovial proliferation in multiple joints, with a characteristic involvement of the small joints of the hands and feet. Diagnosis has long relied on the 1987 modified American College of Rheumatology (ACR) criteria^1^ and more recently the newly proposed ACR/European League Against Rheumatism (EULAR) criteria^2^.

RA is potentially disabling because chronic inflammation of the joints leads to joint destruction. Several predictors of clinical outcome and radiographic progression have been proposed for patients with early RA: radiographic erosions at baseline are highly predictive of further radiographic damage within the next 3 years^3^. Patients with rapid radiographic progression (RRP), defined as structural damage progression of at least 5 points of the van der Heijde-modified Sharp score (mSHS), have poor structural outcome within the next 3 to 8 years of follow-up^4^,^5^. Several biological markers predict radiographic progression: acute-phase reactants (erythrocyte sedimentation rate [ESR] and C-reactive protein [CRP] level)^6^, rheumatoid factor (RF) and anti-cyclic citrullinated (anti-CCP) antibody positivity^7^,^8^,^9^.

Bone erosions resulting from chronic inflammation define both disease severity and insufficient response to disease-modifying anti-rheumatic drugs (DMARDs) and/or biologic therapy. DMARDs and biologic therapy can protect against most radiographic-evidenced damage, but some patients still show structural progression with treatment, which highlights the unmet need for additional markers of structural disease severity and bone-specific targeted treatments.

The Wnt signaling pathway plays a crucial role in bone homeostasis^10^. In the “canonical Wnt signaling pathway” (involving Wnt and β-catenin), Wnt binds the Frizzled receptor and induces low-density lipoprotein receptor-related protein 5/6 (LRP-5/6) phosphorylation, which activates Dishevelled, which in turn represses glycogen synthase kinase 3. This cascade releases axin from its complex with β-catenin and allows β-catenin to translocate to the nucleus. The consequence is the induced expression of various target genes encoding proteins that control osteoblastic differentiation, apoptosis inhibition and other proteins involved in Wnt signaling. Extracellular proteins inhibiting this pathway include sclerostin (SOST) and Dickkopf-related protein 1 (DKK-1)^11^.

SOST is a glycoprotein quite exclusively secreted by mature osteocytes^12^. It travels through the dendritic processes to the bone surface and inhibits osteoblastogenesis by inhibiting Wnt/β-catenin canonical signalling after binding to LRP-5/6. Overexpression of human SOST in mice leads to osteopenia^11^ and loss-of-function mutation leads to van Buchem disease or increased bone density with sclerosteosis^13^,^14^,^15^. Recently, Ardawi *et al*. reported increased expression of serum SOST with age in women^16^. A recently published phase 2 trial suggest that romosozumab, an anti-SOST monoclonal antibody, increased bone mineral density and bone formation and decreased bone resorption in women with low bone mineral density^17^.

DKK-1 was first described as a key regulator of embryonic development because germline deficiency of DKK-1 resulted in lethality, with lack of development of head and limbs^18^. Further studies suggested that DKK-1 is also involved in bone homeostasis. Mice heterozygous for an inactivating mutation of DKK-1 showed high bone mass^19^. In humans, DKK-1 level was higher in myeloma patients with than without bone osteolytic lesions^20^. The same authors reported that myeloma cells expressed high levels of DKK-1, which suggested that inhibition of Wnt signalling was associated with osteolytic lesions.

Therefore, SOST and DKK-1 may be key inhibitors of systemic bone formation, but their involvement in local bone resorption is largely unknown.

Several animal models of RA have helped to decipher the potential role of SOST and DKK-1 in bone erosions. Recently, antibodies directed against SOST were found to enhance bone repair in human tumour-necrosis-factor transgenic (hTNFtg) mice with inflammatory arthritis^21^. This mouse model of RA is characterized by both local and systemic bone loss. SOST antibodies could completely arrest the progression of bone erosion and, with TNF inhibition, induce significant regression of cortical bone erosions. In humans, a pilot study of 22 RA patients suggested increased levels of serum SOST as compared with controls^22^.

The potential role of DKK-1 in subchondral bone erosions in RA was first assessed in animal models. In hTNFtg mice, DKK-1 blockade inhibited bone erosion, which strongly supported the potential role of Wnt signalling in the structural damage observed in RA^23^. Increased serum DKK-1 level was reported in 3 mouse models of RA: hTNFtg mice, mice with collagen-induced arthritis and mice with glucose-6- phosphate isomerase (GPI)-induced arthritis^23^.

Diarra *et al*. assessed DKK-1 level in serum and in the synovium of a small cohort of RA patients with disease duration >1 year and with active disease leading to TNF-blocker therapy (infliximab, 5 mg/kg)^23^. DKK-1 level was higher within the synovium of RA patients than osteoarthritis patients. DKK-1 expression was found in synovial fibroblasts, synovial microvessels and chondrocytes. In addition, functional serum DKK-1 level was higher in RA patients than healthy subjects and was correlated with disease activity assessed by the Disease Activity Score in 28 joints (DAS28)^23^. Daoussis *et al*. observed significantly decreased DKK-1 serum levels with TNF-antagonist treatment in RA patients^24^.

Data assessing the role of SOST and DKK-1 in humans are thus limited and mostly rely on a limited number of patients, which precludes any definite conclusions in this field. Moreover, these studies have quantified functional DKK-1 corresponding to its capacity to bind LRP-6. Data regarding the quantification of free DKK-1 in RA are lacking, as is information on whether DKK-1 or SOST level can predict structural damage, a key issue in RA. We aimed to assess these questions in a large French prospective cohort of early RA patients.

## Results

### Patients with early RA and controls

Among patients from the ESPOIR cohort, 694 fulfilled the ACR/EULAR criteria for RA after 2 years of follow-up. The main characteristics are in Table 1. The mean age was 48.5 ± 12.3 years, 78.2% were female and the mean disease duration from the onset of symptoms to referral to the rheumatologist was 74.8 ± 76.6 days, which corresponded to early RA. Among these patients, 502 (72%) were exposed to a synthetic DMARD (methotrexate, sulphasalazine or leflunomide) within the first 2 years of follow-up and 84 (12%) to a biologic agent: TNF blocker (n = 83) and anakinra (n = 1). TNF blockers were occasionally prescribed as first-line treatment during the inclusion phase of ESPOIR cohort (2002–2005), according to national guidelines in France for treatment of early RA. Among patients receiving biologic agents, for 4, treatment was withdrawn during year 1 of follow-up, 45 started the treatment during year 1 of follow-up and were still receiving a TNF blocker after 2 years of follow-up, and 35 started the treatment during year 2 of follow-up. The 60 sicca controls were age- and gender-matched with RA patients from ESPOIR cohort (mean age 49.0 ± 11.3; 85% female). The age of the 453 healthy controls (47.5% females) from the Variété cohort ranged from 18 to 79 years.

### Decreased expression of SOST among patients with early RA

**Figure 1:**
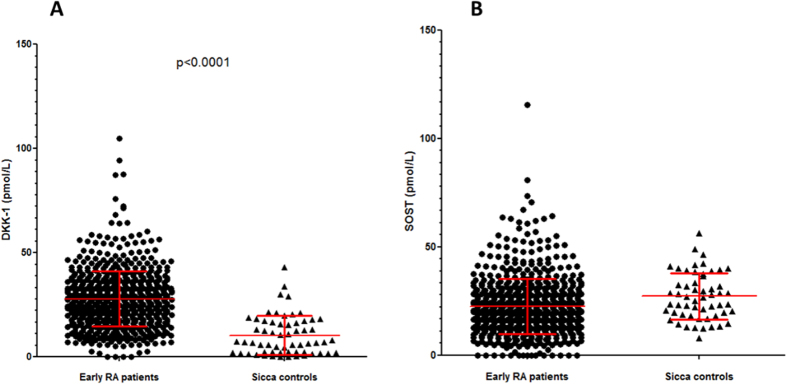
Increased expression of DKK-1 and decreased expression of SOST among patients with early rheumatoid arthritis (RA). Sandwich ELISA or DKK-1 and SOST levels among patients with early RA from the ESPOIR cohort (n = 694) and age- and gender-matched controls (n = 60). (A) SOST level (p = 0.001) (parametric unpaired t-test). (B) DKK-1 level (p < 0.0001) (parametric unpaired t-test). Data are mean (bars) ± interquartile range (whiskers). Each dot represents one patient.

Mean serum SOST levels were significantly lower for RA patients than sicca controls (22.8 ± 15 vs. 27.14 ± 10.7 pmol/L; P = 0.001; Fig. 1B). SOST level for RA patients was significantly correlated with age, weight, height, and body mass index (BMI) (Table 2). Mean SOST level was higher for males than females (25.8 ± 20.7 vs. 21.9 ± 12.8 pmol/L; P = 0.004). SOST level was not associated with any RA activity parameters such as structural damage at baseline or structural progression within the first 2 years of follow-up (data not shown).

We assessed the correlation between SOST and TNF-α levels and other pro-inflammatory factors (IFNγ, IL-1b, IL-2, IL-17, IL-6, MCP-1) or anti-inflammatory cytokines (IL-1Ra, IL-10 and IL-4). SOST and IL-2 levels were slightly and negatively correlated (r = −0.08; p = 0.03). SOST level was not correlated with other studied cytokines. On multivariate analysis adjusted for age, weight, and IL-2 level, only age remained significantly associated with SOST serum levels (p < 0.0001).

### Increased expression of DKK-1 among patients with early RA

As compared with SOST serum levels, mean DKK-1 serum levels were significantly higher in RA patients than sicca controls (28 ± 13.2 vs. 10.3 ± 9.4 pmol/L; P < 0.0001) (Fig. 1a). Serum DKK-1 level did not differ between male and female healthy controls (Suppl. Fig. 2A). DKK-1 serum levels were stable by age among males (Suppl. Fig. 2B) and slightly increased after age 40 years among females (Suppl. Fig. 2C). Indeed, the expression was increased with menopausal status of females (p = 0.0087; Suppl. Fig. 2D), as was recently reported^25^. The stability of free DKK-1 levels by age and gender among these 453 healthy controls strengthens the validity of the increased expression of DKK-1 in patients with early RA as compared with the 60 age- and gender-matched sicca controls.

### Factors associated with increased DKK-1 serum levels at baseline

**Figure 2:**
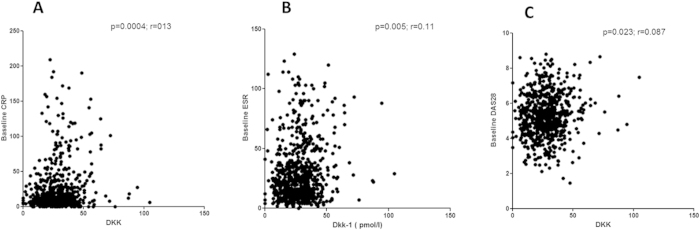
Correlations between serum DKK-1 level and RA activity factors. (**A**) C-reactive protein (CRP) level. (**B**) Erythrocyte sedimentation rate (ESR). (**C**) Disease Activity in 28 joints.

**Figure 3:**
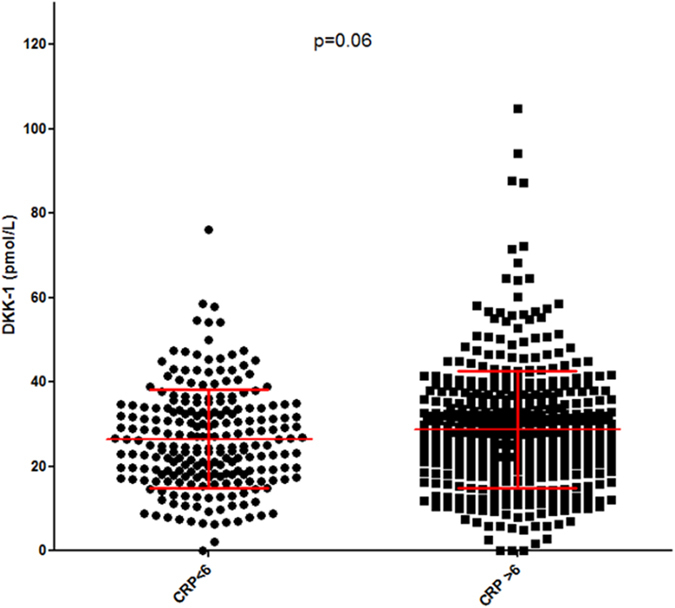
Impact of CRP level on serum DKK-1 level. DKK-1 level (pmol/L) in patients early RA patients with normal vs increased CRP level (threshold 6 mg/L; P_trend_ = 0.06).

**Figure 4:**
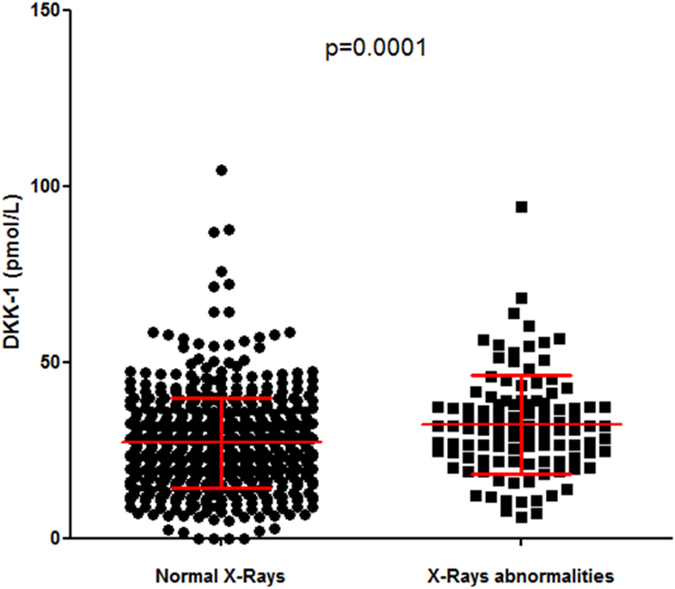
DKK-1 serum levels by typical erosive lesions at baseline. DKK-1 level (pmol/L) in patients with normal X-rays (n = 590) and typical erosive lesions (n = 104) at baseline. Data are mean ± SD. (unpaired t test).

On univariate analysis, DDK-1 level was significantly correlated with CRP level (r = 0.13; p < 0.0004; Fig. 2A), ESR (r = 0.11; p = 0.005) (Fig. 2B), patient global assessment of disease (PGA) (r = 0.08; p = 0.046) and DAS28 (r = 0.087; p = 0.023) (Fig. 2C). We also wondered whether increased level of DKK-1 among RA patients as compared with controls was exclusively related to systemic inflammation, so we compared DKK-1 levels by CRP levels (normal vs increased; threshold 6 mg/L) for RA patients. Patients with CRP level >6 mg/L showed a trend for increased DKK-1 level, but mean DKK-1 level was still higher for patients with normal than increased CRP level (28.7 ± 13.9 vs 26.5 ± 11.7 pmol/L; P_trend_ = 0.06) (Fig. 3). In addition, mean DKK-1 level was significantly higher for patients with than without typical erosions related to RA at baseline (n = 110 vs n = 584) (32.3 ± 14.0 vs 27.2 ± 12.9 pmol/L; P = 0.0001) (Fig. 4).

Increased DKK-1 level was not associated with other patient characteristics (gender, anti-CCP antibody positivity). We found a small but non-significant increase in mean DKK-1 level among ever-smokers as compared with non-smokers (28.9 ± 13.0 vs 27.2 ± 14.1 pmol/L; P_trend_ = 0.09). All patients included in the cohort were not taking corticosteroids or biologic therapy at baseline. NSAIDs and DMARDs did not have any impact on DKK-1 serum levels (data not shown).

The level of DDK-1 was significantly but poorly correlated with level of SOST among patients with early RA (r = 0.10; p = 0.006) (Suppl. Fig. 3). We also assessed the level of correlation between DKK-1 and several cytokines and chemokines quantified at baseline in ESPOIR cohort. This exploratory analysis showed few significant correlations (Suppl. Table 1), MCP-1 and IL-6 being the most strongly correlated (MCP-1: r = 0.19; p < 0.0001 and IL-6: r = 0.16; p < 0.0005) (Suppl. Table 1).

On multivariate analysis adjusted for factors with significant or possible association with DKK-1 level on univariate analyses (DAS28, PGA, smoking status, and levels of several cytokines), only CRP level, presence of typical erosions related to RA and MCP-1 level remained associated with DKK-1 level (Table 3).

### DKK-1 is a marker of structural severity at baseline

In total, 110 RA patients had typical erosive lesions at baseline. Several factors were significantly associated with these early structural lesions among RA patients: anti-CCP antibody positivity (P = 0.0002), gender (females), age (P = 0.04), increased CRP level or ESR (P = 0.016 and P = 0.005, respectively), and DKK-1 and IL-6 levels (P = 0.0001 and P < 0.0001, respectively). Structural lesions were associated but not significantly with PGA (P = 0.09) and DAS28 (P = 0.09). On multivariate analysis, including all these parameters, only older age (P = 0.02), anti-CCP antibody positivity (P = 0.05), DKK-1 level (P = 0.015) and IL-6 level (P = 0.006) remained significantly associated with baseline erosions.

To better delineate the respective effect of DKK-1 and IL-6 levels on structural severity at baseline, we included DKK-1 and IL-6 levels as quartiles in the model. The main risk of structural severity at baseline was associated with positive anti-CCP antibodies (adjusted OR [aOR] 1.6, 95% CI [1.02–2.51]; P = 0.04); the upper versus lowest quartile (>13.8 vs <0.5 ng/mL) of IL-6 level (aOR 1.85, 95% CI [0.97–3.5]; P = 0.02); and the upper versus lowest quartile (>34.8 vs <19.2 pmol/L) of DKK-1 level (aOR 2.71, 95% CI [1.40–5.26]; P = 0.0045).

### DKK-1 is a predictor of structural progression

Baseline serum DKK-1 level was associated with structural progression within the first 2 years of follow-up. Mean baseline DKK-1 level was higher among RA patients with than without radiological progression within the first year of follow-up (29.6 ± 13.3 vs 26.63 ± 12.4 pmol/L) (p = 0.0084) and within the first 2 years of evolution (29.5 ± 13.1 vs 26.0 ± 11.7 pmol/L; p = 0.0027). Other factors associated with structural progression on univariate analyses were anti-CCP antibody positivity (p < 0.0001), FR positivity (p = 0.0003), MCP-1 level (p = 0.012), IL-6 level (p = 0.04), baseline erosion (p = 0.001), and exposure to biologic therapy within the first 2 years of follow-up (p = 0.008).

**Figure 5:**
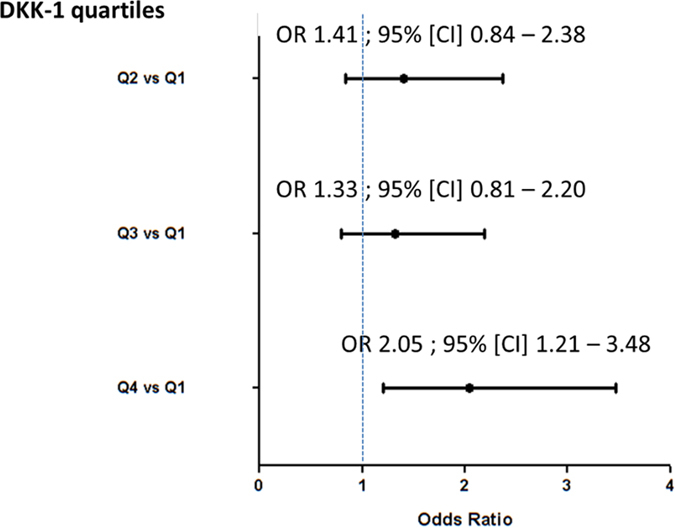
Risk of structural progression in early RA patients within the first 2 years of follow-up by quartiles (Q) of serum DKK-1 level. DKK-1 serum levels among patients with early RA show a Gaussian distribution. Q1, <19.2; Q2, 19.2–27.3; Q3, 27.3–34.8, and Q4 >34.8 pmol/L.

On multivariate analysis (Table 4), DKK-1 level remained associated with radiographic progression in a model including the previously identified predictors of structural severity. In this model, including DKK-1 level at baseline, MCP-1 but not IL6 level was still significantly associated with structural progression within the first 2 years of follow-up, with a trend for exposure to a biologic therapy within the first 2 years (p_trend_ 0.08). When considering DKK-1 distribution by quartiles, DKK-1 level in the upper quartile (>34.8 pmol/L) was associated with risk of structural progression (OR 2.05, 95% CI [1.21–3.48]) as compared with the lowest quartile (<19.2 pmol/L; Fig. 5 and Table 5). When comparing the main patients characteristics according to DKK-1 quartile, we did not find any difference between the lower and upper quartile regarding demographic characteristics, disease activity, antibody status, but we confirmed a higher CRP level among patients of the upper quartile (28.1 ± 36.8 vs 15.9 ± 32.5, p = 0.0012), and a higher frequency of typical RA erosions (23.6% vs 8.7%, p = 0.0002). The percentage of patients with typical erosions was increased according to DKK-1 quartiles: 15/172 (8.7%) (Q1), 28/174 (16.7%) (Q2), 26/174 (14.9%) (Q3), and 41/174 (23.6%) (Q4). To delineate the relative weight of DKK-1 and other factors known to be associated with structural damage, we included quartiles of DKK-1 level in multivariate analysis and confirmed that the upper quartile of DKK-1 level was associated with increased risk of structural progression in the same range as other recognized risk factors of poor structural outcome (i.e., anti-CCP antibody positivity and baseline erosions; Table 5). On sensitivity analysis, as compared with IL-6 level, IL-6 as a binary variable (detectable/non-detectable) was associated with radiographic progression within the first 2 years (P = 0.002).

In total, 106 patients showed rapid radiographic progression (RRP). Factors significantly associated with RRP were anti-CCP antibody positivity (p < 0.0001), FR positivity (p = 0.0001), baseline erosion (p = 0.0015), increased ESR (p = 0.008) or CRP level (p = 0.0002), exposure to synthetic DMARDs within the first 2 years of follow-up (p = 0.002), TNFα level (p = 0.015), and IL-6 level (p = 0.02). DKK-1 level was not significantly associated with RRP. Nevertheless, on multivariate analysis, only anti-CCP antibody positivity and increased CRP level at baseline remained significantly associated with RRP (p = 0.0006 and p = 0.045, respectively).

## Discussion

We found increased serum DKK-1 level in a large cohort of early RA patients as compared with controls. This increased expression was associated with disease activity, elevated acute-phase reactants and bone erosions at baseline. Of note, increased DKK-1 level at baseline was an independent predictor of structural progression after 2 years of follow-up, so DKK-1 might be an interesting new structural biomarker in early RA.

The strength of the present study is the large number of patients assessed within the ESPOIR cohort, their long-term prospective follow-up with iterative radiological evaluation, which allowed for studying the role of DKK-1 as a predictive biomarker of structural damage. The other advantage of the ESPOIR cohort is that the quantification of DKK-1 at baseline was not distorted by treatments, as was previously reported for TNF blockers^23^,^24^.

DKK-1 level was greatly increased among early RA patients as compared with controls. This observation agrees with previous reports showing functional DKK-1 level also increased in RA patients^23^. Our study featured many quality controls and we evaluated the impact of age and gender of DKK-1 serum levels from healthy controls. These experimental data attest to the internal validity of the results.

However, we did not assess the effect of high DKK-1 level on systemic bone loss. In fact, bone mineral density (BMD) measurements were not planned in ESPOIR cohort. Nevertheless, according to the already well-defined role of DKK-1 in bone homeostasis, patients with increased serum DKK-1 level are at risk of osteopenia or osteoporosis. This point needs to be further assessed in a cohort of early RA patients, who are free of corticosteroids, and with a prospective evaluation of their BMD.

SOST serum levels were mostly associated with demographic factors in early RA and not associated with any inflammatory or activity factors of the disease. This observation should not weaken the potential importance of SOST level in systemic and/or local bone homeostasis in RA. In fact, SOST is exclusively produced by mature osteocytes as compared with DKK-1, which is ubiquitously produced by many cell types. Therefore, serum SOST level may not reflect its local expression in subchondral bone. Moreover, recent results in hTNFtg mice with RA, in which anti-SOST antibodies completely arrested the progression of bone erosion, also sustains the importance of SOST in RA^21^.

We found DKK-1 level an important marker of structural severity at baseline, particularly for patients with levels within the upper quartile. The factors significantly associated with DKK-1 level were related to biological inflammation and included DAS28, ESR, and CRP level. DAS28 is a composite index that assesses the activity of the disease and is composed of 4 factors: number of tender and swollen joints (not associated with DKK-1 serum levels), patient global assessment of disease (slightly associated with DKK-1 serum levels) and ESR, the most strongly associated factor among DAS28 factors. Other markers of biological inflammation, such as IL-6 level and to a lesser extent TNF-α level, were slightly but significantly associated with DKK-1 level. These results agree with previous studies showing that TNF-α level (i.e., inflammation) is a potent inducer of DKK-1 expression^23^,^26^.

The correlation between serum levels of DKK-1 and MCP-1 has never been reported in RA. MCP-1 is a member of the C-C chemokine family and is a potent chemotactic factor driving monocytes, T cells, and dendritic cells to sites of tissue inflammation. Monocytes/macrophages are the main source of MCP-1. MCP-1 could be considered a surrogate marker of inflammation, which explains the correlation between DKK-1 and MCP-1 levels we observed. Nevertheless, we did not find a positive correlation between CRP and MCP-1 levels, which suggests that mechanisms inducing MCP-1 expression are independent of inflammation. Moreover, the association of DKK-1 and MCP-1 levels was greater than DKK-1 and TNF-α levels. Finally, even among patients with normal CRP level, DKK-1 level was still highly increased. These observations suggest that inflammation accounted for only part of the increase in DKK-1 and MCP-1 levels and that an alternative biologic mechanism might induce their expression. Indeed, He *et al*. reported that in myeloma cells, the expression of both DKK-1 and MCP-1 was potently induced by constitutive activation of p38 mitogen-activated protein kinase (MAPK) signaling^27^. p38-induced DKK-1 level inhibited osteoblastogenesis (a well-known function of DKK-1) and also acted synergistically with MCP-1 to induce osteoclast differentiation and bone resorption through RANKL secretion from bone-marrow stromal cells^27^. Thus, a constitutive but not exclusively TNF-induced activation of p38 MAPK, as previously reported in mice^22^, in the rheumatoid joint could locally induce DKK-1 and MCP-1 and synergistically lead to bone erosions through mechanisms similar to those observed in myeloma cells.

Increased expression of DKK-1 in RA could also be genetically controlled in part, as was suggested by de Rooy *et al*. in a large cohort of RA patients in which 3 DKK-1 single nucleotide polymorphisms (SNPs) were associated with structural progression. Presence of one of the SNPs (rs1896368) was slightly associated with increased levels of functional DKK-1 among patients carrying the at-risk rs1896368-GG genotype^28^. Nevertheless, we genotyped rs1896368 in ESPOIR cohort and did not replicate these findings^29^. The contribution of other polymorphisms located within DKK-1 locus and involved in DKK-1 regulation cannot be excluded.

DKK-1 level was not associated with RRP. In fact, factors associated with RRP on univariate analyses were mainly those associated with inflammation – CRP level, ESR, and TNF-α and IL-6 levels – as was previously demonstrated in the ESPOIR cohort^5^,^30^. These previous studies, with different time points and definitions of progression and not including DKK-1 data, showed that IL-6 but not CRP level was an independent factor of structural progression. However these 2 factors are undoubtedly linked. In our study, multivariate analysis retained only increased level of CRP or anti-CCP antibody positivity at baseline associated with RRP, which demonstrates that cytokines might have little association with rapid structural progression. Nevertheless, free serum DKK-1 level may be unsuitable for identifying RRP because of its imperfect reflection of locally produced DKK-1. DKK-1 levels in synoviocytes from patients with RRP would be interesting to assess. Moreover, our study exclusively assessed baseline levels of serum DKK-1. The area under the receiver operating characteristic curve of DKK-1 within the first 2 years of follow-up might be more informative to identify RPP, especially for patients with persistently high levels of serum DKK-1 despite DMARDs and/or biologic treatments.

This study demonstrates for the first time that DKK-1 may be a strong determinant of structural damage at baseline among patients with early RA and may be an independent predictor of slow structural progression defined by increased mSHS >1 within 2 years of follow-up. Patients with high DKK-1 level (upper quartile, >34.8 pmol/L) showed increased risk of structural progression in the same range as risks attributed to anti-CCP antibody positivity or baseline erosions. Including DKK-1 serum quantification in clinical practice for early RA patients may be beneficial to identify patients with potential poor structural outcome who may need close monitoring for development of early bone erosions and rapid adjustment of DMARDs and/or biologic therapy. DKK-1 may be a promising therapeutic target in RA. Anti-DKK-1 antibodies have been successfully used as therapy in mouse models of RA^23^ and were able to revert bone erosions. In human diseases, anti-DKK-1 antibodies are under investigation for smoldering myeloma (phase 2 trial) but not yet RA. Our findings and those from animal models of RA in which anti-DKK-1 antibodies have been successfully used provide robust arguments to further evaluate DKK-1 blockade in RA to try to limit the structural damage in RA and even to heal erosions in patients with overt structural damage.

## Patients and Methods

### Patients and controls

#### Patients

In total, 813 patients were included in the ESPOIR cohort (Etude et Suivi des Polyarthrites Indifférenciées Récentes [“Study and follow-up of early undifferentiated polyarthritis”]). Patients had to have inflammatory arthritis involving at least 2 swollen joints, lasting from 6 weeks to 6 months, with suspected or confirmed diagnosis of RA. The main characteristics of patients at baseline were previously reported^31^. Overall, 694 patients fulfilled the 2010 ACR/EULAR criteria after 2-year follow-up. Patients were excluded if the referring physician considered another defined inflammatory rheumatic disease. To be included in the cohort, patients had to be free of any steroids and DMARDs (except within the 15 days before inclusion for DMARDs only). Clinical, biological and radiological data are prospectively collected throughout follow-up (i.e., every 6 months during the first 2 years, and every year thereafter). The follow-up is scheduled for at least 15 years from December 2002. Fourteen regional centers in France participated in patient inclusion.

#### Patient’s clinical and biological assessments

Clinical variables assessed included total joint count for tenderness and swelling, DAS28^32^, patient global assessment of disease (PGA), and the Health Assessment Questionnaire (HAQ) score^33^. Laboratory variables included ESR (mm/h), CRP, IgM and IgA RF (ELISA for both, Menarini France, both positive if >9 UI/ml), and anti-CCP antibodies (ELISA, DiaSorin, France; positive if >50 U/ml).

#### Structural assessment

Patients with early RA included in the cohort undergo radiographic evaluation every 6 months during the first 2 years of follow-up, which allows for studying factors associated with structural progression. Radiographs of the hands and feet (antero-posterior views) were collected in the radiography coordinating center. X-rays obtained at baseline and at 1 and 2 years of follow-up were read in a standardized way. All sets of X-rays were read with information on the chronology of the films to improve sensitivity to change by a trained investigator blinded to clinical evaluation (GT; intra-reader correlation coefficient 0.97; GT and VDP inter-observer correlation 0.93 for the mSHS; the smallest detectable change was 1)^5^. Structural damage was assessed qualitatively by the presence of typical RA erosions according to their location and aspect and rated according to the mSHS^34^ on radiographs of both hands and wrists, and feet. The mSHS scoring method includes, in each hand, 16 areas for erosions and 15 areas for joint space narrowing, and, in each foot, 6 areas for erosions and 6 areas for joint space narrowing. The maximal total erosion score (hands and feet) is 280. The maximal total narrowing or (sub)luxation score (hands and feet) is 168. The total score ranges from 0 to 448. Radiographic progression was defined as an increase in mSHS >1, assessed between baseline and the end of years 1 and 2 of follow-up. This threshold of 1 was chosen as the smallest detectable difference (SDD), corresponding to the difference being above the measurement error. RRP was defined as an increase in mSHS >10 within the first 2 years of follow-up (i.e., 5 points per year) as previously proposed in ESPOIR cohort^35^. We also considered the “typical RA-erosion” criteria defined according to the physician judgment, as previously proposed for the diagnosis of RA in the ACR/EULAR classification criteria^36^.

#### Controls

A first group of controls were patients with sicca symptoms without any autoimmunity feature and were referred to the Rheumatology Department of Bicêtre Hospital for a diagnostic procedure. These 60 controls were age- and gender-matched to a random sample of 60 RA patients from the ESPOIR cohort.

A second group of 453 healthy controls, in whom DKK-1 serum levels were assessed, were from the Variété cohort, a transversal, non-interventional French national cohort of healthy volunteers that establishes normative data for insulin-like growth factor 1 (IGF-1) and other hormones in the general population (ClinicalTrials.gov identifier: NCT01831648). This cohort is a large random selection of subjects from the general population including representation from all age groups (about 100 subjects for each decade age range). Subjects with medical conditions and medications that may affect IGF-I measurement are excluded. A total of 974 healthy subjects have been recruited in 10 centres in France. Each subject undergoes clinical examination. Personal medical history is recorded and gonadal status evaluated.

#### Ethics

The protocols for the ESPOIR and Variété cohorts were performed in accordance with the French guidelines and regulatory authorities. These protocols were approved by the ethics committees of Montpellier University Hospital for the ESPOIR cohort and Hôpitaux Paris-Sud for the Variété cohort. All patients from the cohorts and sicca controls gave their written informed consent for use of their data.

#### Serum DKK-1 and SOST measurements

DKK-1 and SOST serum levels were assessed at baseline in the whole ESPOIR cohort, but all analyses were restricted to the subgroup of patients fulfilling the ACR/EULAR criteria (n = 694). We assessed DKK-1 and SOST serum levels in 60 sicca controls. Because of lack of data on the impact of gender and age on DKK-1 serum levels among the healthy population, we further assessed 453 healthy controls from the Variété cohort for DKK-1 serum levels in a broader range of age than those matched for ESPOIR cohort.

All subjects in the Variété cohort underwent biological standard workup, with 80-ml blood samples taken, serum aliquoted, and frozen and stored at −80 °C before hormone measurements. Serum samples from the ESPOIR cohort were prospectively collected at inclusion from December 2002 to March 2005 and stored in aliquots at −80 °C in the Biological Resources Center at Bichat Hospital (accreditation AFNOR #34457).

Serum levels of SOST and DKK-1 were assessed by sandwich ELISA (Biomedica Medizinprodukte, Vienna, Austria). For DKK-1 quantification, serum samples were diluted 1:4 as recommended by the manufacturer. DKK-1 serum levels from the ESPOIR cohort and 60 age- and gender-matched sicca controls were assessed with the first-generation set of the ELISA kit (Lot F112). DKK-1 serum levels from the Variété cohort were assessed 18 months later with the second-generation set of the ELISA kit (Lot F125). All SOST serum levels from the ESPOIR cohort and 60 age- and gender-matched sicca controls were assessed with the second-generation set of the ELISA kit (Lot Y128). SOST serum levels were not assessed on a larger sample of healthy controls because data were already available in the literature regarding the impact of gender and age on the quantification^16^,^37^.

For DKK-1, results are expressed in picomoles per litre. The conversion to picograms per millilitre was as follows: 1 pmol/L = 28.68 pg/mL.

All serum samples were manually distributed on coated plates, but washing and distribution of the conjugate, substrate and stop solution were automatic with the Evolis microplate system (Bio-rad, Marnes-La-Coquette, France). Optical density was read immediately at 450 nm on the Evolis system.

Several quality controls were performed throughout the study: an internal control for both DKK-1 and SOST levels was provided by the ELISA manufacturer and quantified on each ELISA plate for validation of each experiment. The internal control quantifications were expected to be 3.1 to 5.9 pmol/L for DKK-1 and 40 to 74 pmol/L for SOST. We performed 26 quantifications of the internal control (13 in duplicate), with mean DKK-1 level 4.31 ± 0.4 pmol/L and mean SOST level 57.48 ± 2.9 pmol/L. All experimental results were within the expected ranges and thus validated (Suppl. Fig 1A,B).

Serum providing D.O. >3.5 (>50 mol/L) was diluted 1:2 and re-quantified. We tested 80 serum samples in duplicate and demonstrated no significant variation in quantification (data not shown).

Other cytokines measured in the ESPOIR cohort at baseline with a commercially available multiplex bead immunoassay based on the Luminex platform (Fluorokine MAP Multiplex HumanCytokine Panel, R&D Systems, Minneapolis, MN, USA) included interleukin 1β (IL-1β), IL-1 receptor antagonist (IL1-Ra), IL-2, IL-4, IL-6, IL-10, IL-17, monocyte chemoattractant protein 1 (MCP-1), TNF-α and interferon γ (IFN-γ) as previously described^30^.

### Statistical analysis

All analyses were restricted to the subgroup of 694 patients fulfilling the ACR/EULAR criteria. Categorical variables are reported as number (percentage) and were compared by chi-square test or, as appropriate, Fisher’s exact test. Quantitative variables are reported as mean ± SD or median (range) and were compared by Student *t* test. Factors associated with DKK-1 or SOST level were assessed by correlation analysis with Spearman’s correlation coefficient. All variables with p ≤ 0.10 on univariate analysis were entered into a multivariate linear regression model to identify independent predictors of DKK-1 or SOST levels. To identify factors associated with radiographic progression (defined by increase in mSHS >1 or >10 within the first 2 years of evolution), we compared demographic, clinical, biological and radiographic factors between patients with and without radiographic progression. All variables with p ≤ 0.10 on univariate analysis were entered into a multivariate logistic regression model to identify independent predictors of radiographic progression. For all analyses, *P* < 0.05 was considered statistically significant. Odds ratios (ORs) and 95% confidence intervals were calculated. Statistical analysis involved use of SAS 9.3 (SAS Inst., Cary, NC).

## Additional Information

**How to cite this article**: Seror, R. *et al*. Increased Dickkopf-1 in Recent-onset Rheumatoid Arthritis is a New Biomarker of Structural Severity. Data from the ESPOIR Cohort. *Sci. Rep.* **6**, 18421; doi: 10.1038/srep18421 (2016).

## Supplementary Material

Supplementary Information

## Figures and Tables

**Table 1:** Baseline demographics and disease characteristics of patients with early rheumatoid arthritis (RA) from the ESPOIR cohort.

	**No. of patients with available data**	**Early RA patients n = 694**
Gender (female)	694	543 (78.2%)
Age (years)	694	48.5 (12.3)
BMI (kg/m^2^)	*692*	25.1 (4.6)
Ever-smoker	694	333 (48.0%)
CRP (mg/dl)	*684*	22.8 (34.7)
US-CRP^a^ (mg/dl)	694	20.9 (33.5)
ESR (mm)	*684*	29.9 (24.8)
PGA (/100 mm)	*692*	61.7 (24.6)
Tender joint count (/28)	694	9.4 (7.1)
Swollen joint count (/28)	694	7.9 (5.4)
DAS28	*681*	5.3 (1.2)
Anti-CCP antibodies positive	694	315 (45.4%)
Positive IgM-RF^b^	694	372 (53.6%)
Typical RA erosive change	694	110 (15.7%)
Modified Sharp score	*658*	5.2 (7.4)
Current use of DMARD	694	48 (6.9%)
Current use of oral corticosteroids	694	0 (0.0%)
Current use of oral NSAIDs	694	487 (70.2%)
DKK (pmol/L)	694	28.0 (13.2)
SOST (pmol/L)	694	22.8 (15.0)

Data are mean ± SD or number (%).

^a^ultrasensitive C-reactive protein (CRP) level.

^b^ESR, erythrocyte sedimentation rate; RF: rheumatoid factor (IgM); BMI: body mass index; DAS28: disease activity score in 28 joints; DMARDs: disease modifying anti-rheumatic drugs; NSAIDs: nonsteroidal anti-inflammatory drugs; PGA: patient global assessment of disease; DKK: Dickkopf-1; SOST: sclerostin.

**Table 2:** Correlation between serum SOST level and baseline characteristics of RA patients in the ESPOIR cohort.

**Parameter**	**No. of patients**	**Spearman r**	**p-value**^a^	**p-value**^b^
Age	694	0.25	**<0.0001**	**<0.0001**
Weight	693	0.14	**0.0002**	0.62
Height	993	0.08	**0.03**	0.06
BMI	692	0.12	**0.015**	

^a^Univariate analysis.

^b^Multivariate analysis with BMI excluded.

**Table 3:** Factors associated with high serum DKK level (multivariate analysis).

**Parameter**	**p-value**
DAS28	0.83
PGA	0.13
***CRP level***	***0.03***
***Typical RA erosive change***	***0.009***
Ever-smoker	0.17
IL-1Ra level	0.40
IL-6 level	0.10
***MCP-1 level***	***0.05***
TNFα level	0.89
IL-17 level	0.10
IL-4 level	0.40

Multivariate analysis with a linear model including all variables with p < 0.10 on univariate analysis.

**Table 4:** Factors associated with structural progression defined by an increase of van der Heijde-modified Sharp score (mSHS) >1 within the first 2 years of follow-up (multivariate analysis).

**Parameter**	**Adjusted OR**	**95% CI**	**p-value**
***DKK-1***^a^	1.017	1.001–1.034	***0.04***
***Anti-CCP antibodies***^b^	1.715	1.141–2.579	***0.009***
IL-6 level^c^	1.006	0.994–1.018	0.32
IL-1RA level^c^	1.000	1.000–1.000	0.85
***MCP-1 level***^c^	1.002	1.000–1.004	***0.04***
***Typical erosion***^d^	1.859	1.006–3.434	***0.05***
DMARDs^e^	1.234	0.771–1.974	0.38
Biologic therapy^f^	1.796	0.931–3.466	0.08

OR: odds ratio; 95% CI, 95% confidence interval.

^a^estimate for an increase of 1 pmol/L in DKK-1 serum level.

^b^patients positive versus negative for anti-CCP antibodies.

^c^estimate for an increase of 1 ng/ml in IL-6, IL-1RA or MCP-1 serum levels.

^d^typical erosion at baseline, presence versus absence.

^e^exposure to a synthetic DMARD within the first 2 years of follow-up.

^f^exposure to a biologic therapy within the first 2 years of follow-up.

**Table 5:** Factors associated with structural progression defined by an increase in mSHS >1 within the first 2 years of follow-up with quartiles of DKK-1 serum levels included in the model (multivariate analysis).

**Parameter**	**Adjusted OR**	**95% CI**	**p-value**
DKK-1 level			
Q2 vs Q1	1.31	0.77–2.23	0.95
Q3 vs Q1	1.19	0.71–2.0	0.60
***Q4 vs Q1***	2.05	1.21–3.48	***0.05***
***Anti-CCP antibodies***	1.68	1.13–2.49	***0.01***
***Typical erosions***	1.94	1.06–3.55	***0.03***
Biologic therapy	1.86	0.98–3.55	***0.06***

Q1, <19.2; Q2, 19.2–27.3; Q3, 27.3–34.8, and Q4 >34.8 pmol/L.
