# Supplementary material for: Increased Dickkopf-1 in Recent-onset Rheumatoid Arthritis is a New Biomarker of Structural Severity. Data from the ESPOIR Cohort
Source: Sci Rep. 2016 Jan 20;6:18421. doi: 10.1038/srep18421 (PMC4726234; doi:10.1038/srep18421)
Supplement: Supplementary Information [file srep18421-s1.doc]

**Increased Dickkopf-1 in Recent-onset Rheumatoid Arthritis is a New Biomarker of Structural Severity. Data from the ESPOIR Cohort**

**Raphaèle Seror**^1,2^, Saida Boudaoud^2^, Stephan Pavy^1^, Gaetane Nocturne^1,2^,Thierry Schaeverbeke^3^, Alain Saraux^4^, Philippe Chanson^5,6^, Jacques-Eric Gottenberg^7^, Valérie Devauchelle-Pensec^4^, Gabriel J. Tobón^4^, Xavier Mariette^1,2^,and Corinne Miceli-Richard^1,2^.

Supplementary file


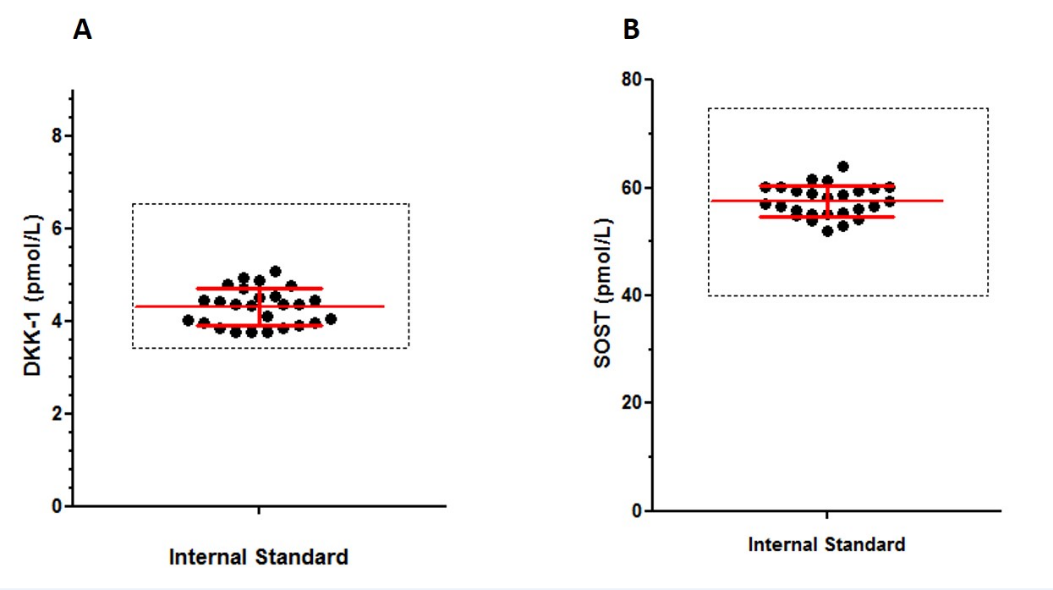


**Supplementary Figure 1. Internal standard quantifications for DKK-1 and SOST.**

The internal controls for DKK-1 and SOST quantification were provided by Biomedica and expected to be 3.1–5.9 pmol/L for DKK-1 and 40–74 pmol/L for SOST (represented by the dotted rectangles). In total, 26 quantifications were performed (13 in duplicate). (A) DKK-1 internal control, mean 4.31±0.4 pmol/L. (B) SOST internal control, mean 57.48±2.9 pmol/L.

**
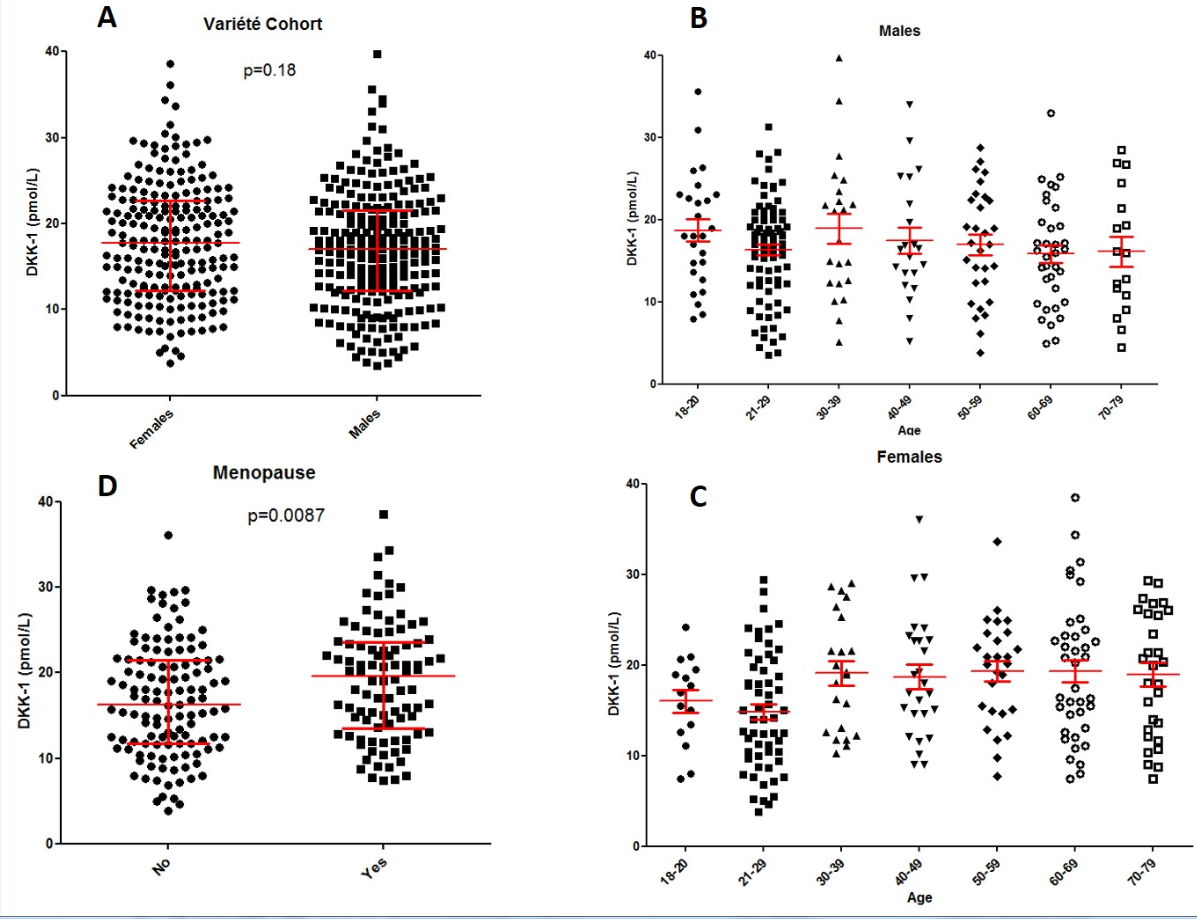
**

**Supplementary Figure 2. ELISA quantification of DKK-1 level among healthy controls from the Variété cohort.** (A) Females (n=220) and males (n=246) (p=0.22). Data are mean (interquartile range). (B) DKK-1 quantification among healthy males by decade age groups (p=0.68; Kruskal-Wallis test. Data are mean±SD. (C) DKK-1 quantification among healthy females by age groups (p=0.013; Kruskal-Wallis test). Data are mean±SEM. (D) DKK-1 quantification among healthy females by menopausal status (yes/no) (p=0.012; unpaired t test). Data are mean±SEM.


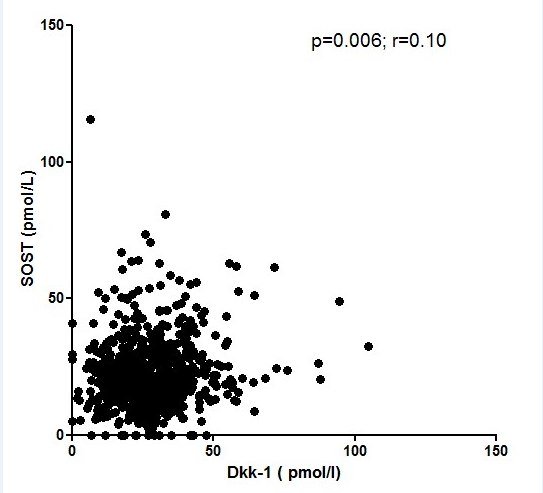


**Supplementary Figure 3. Correlation between DKK-1 and SOST in ESPOIR cohort.**

**Suppl. Table 1**. Correlation between DKK-1 and cytokines and chemokines all quantified at baseline in ESPOIR cohort.

|  | N | r (CI 95 %)^a^ | p-value |
| --- | --- | --- | --- |
| IL-1RA | 630 | 0.11 (0.03 – 0.18) | 0.0064 |
| **IL-6** | **630** | **0.16 (0.08 – 0.23)** | **<.0005** |
| IL-10 | 630 | 0.006 (-0.07 – 0.084) | 0.86 |
| **MCP-1** | **630** | **0.19 (0.11 – 0.26)** | **<.0001** |
| IL-4 | 630 | 0.084 (0.007 – 0.16) | 0.03 |
| IL-17 | 630 | 0.07 (-0.002 – 0.15) | 0.06 |
| IFNγ | 630 | -0.04 (-0.12 – 0.03) | 0.28 |
| TNFα | 629 | 0.08 (0.005 – 0.16) | 0.04 |
| IL-1b | 630 | 0.02 (-0.06 – 0.099) | 0.58 |
| IL-2 | 630 | -0.01 (-0.09 – 0.07) | 0.78 |

^a^: Spearman correlation coefficient (confidence interval 95%)
